# Supplementary material for: Multi-omics analyses reveal that the gut microbiome and its metabolites promote milk fat synthesis in Zhongdian yak cows
Source: PeerJ. 2022 Dec 2;10:e14444. doi: 10.7717/peerj.14444 (PMC9744170; doi:10.7717/peerj.14444)
Supplement: Supplemental Information 13 [file peerj-10-14444-s013.zip › Web_Report/Web_Report_directory_readme.html]

/share02/bioCloud/cloud/publish\_compute\_task\_shell/u4359N4/pos\_metab\_20211201114359682/Web\_Report/Web\_Report\_directory\_readme.html

结果目录说明

## 结果目录说明

Web\_Report

- Data\_assess 数据评估结果
  - group\_info.txt 样本分组信息
  - metabolites\_intensity 代谢物定量结果
    - All\_metabolite\_profiles.xls 所有鉴定代谢物定量文件
    - metabolites\_exp.xls 鉴定到代谢物名称的定量文件
    - metabolites\_full\_table.xls 带有代谢物注释信息的定量文件
  - correlation\_analysis 样本相关性结果
    - All\_cor.pdf 样本相关性热图
    - All\_cor.png 样本相关性热图
    - All\_cor.xls 样本相关性文件
  - heatmap 所有样本聚类热图结果
    - All\_heatmap.pdf 所有样本聚类热图
    - All\_heatmap.png 所有样本聚类热图
    - All\_heatmap.xls 所有样本聚类热图作图文件
  - PCA 所有样本主成分分析结果
    - All\_pca3D.pdf 所有样本PCA分析三维图
    - All\_pca3D.png 所有样本PCA分析三维图
    - All\_pca.pdf 所有样本PCA分析二维图
    - All\_pca.png 所有样本PCA分析二维图
    - All\_pca\_loadings.xls 所有样本PCA分析载荷值文件
- Metabolites\_annotation 所有代谢物数据库注释结果
  - KEGG 所有代谢物KEGG数据库注释结果
    - meta\_kegg\_anno\_categroy.xls 所有代谢物KEGG通路统计结果
    - meta\_kegg\_anno.xls 所有代谢物KEGG注释结果
    - meta\_kegg\_anno.png 所有代谢物KEGG通路top20柱形图
    - All\_kegg\_map 所有代谢物通路图注释
      - ko\*.html 所有代谢物通路图
      - ko\*.png 所有代谢物通路图
  - HMDB 所有代谢物HMDB数据库注释结果
    - meta\_hmdb\_anno.xls 所有代谢物HMDB数据库注释表格
    - meta\_hmdb\_anno\_categroy.xls 所有代谢物HMDB数据库分类表格
    - meta\_hmdb\_anno.pdf 所有代谢物HMDB数据库分类top20柱形图
  - Lipidmaps 所有代谢物Lipidmaps数据库注释结果
    - meta\_lipidmaps\_anno.xls 所有代谢物Lipidmaps数据库注释表格
    - meta\_lipidmaps\_anno\_categroy.xls 所有代谢物Lipidmaps数据库分类结果
    - meta\_lipidmaps\_anno.png 所有代谢物Lipidmaps数据库分类top20柱形图
- Diff\_analysis 各个分组差异分析结果
  - Diff\_KEGG\_anno\_stat.xls 差异代谢物KEGG注释统计结果
  - \*\_vs\_\* 差异分组分析结果
    - \*\_vs\_\*.all.xls 未经过筛选的差异分析结果
    - \*\_vs\_\*.diff\_final.xls 经过筛选的差异分析结果
    - \*\_vs\_\*\_diff\_heatmap.pdf 差异代谢物聚类热图(进行样本聚类)
    - \*\_vs\_\*\_diff\_heatmap.png 差异代谢物聚类热图(进行样本聚类)
    - \*\_vs\_\*\_diff\_heatmap\_name.pdf 差异代谢物聚类热图(进行样本聚类且添加代谢物名称)
    - \*\_vs\_\*\_diff\_heatmap\_nonclustered\_samples.pdf 差异代谢物聚类热图(不进行样本聚类)
    - \*\_vs\_\*\_diff\_heatmap\_nonclustered\_samples.png 差异代谢物聚类热图(不进行样本聚类)
    - \*\_vs\_\*\_diff\_heatmap\_nonclustered\_samples\_name.pdf 差异代谢物聚类热图(不进行样本聚类且添加代谢物名称)
    - \*\_vs\_\*\_OPLS\_DA.pdf 差异分组OPLS-DA得分图
    - \*\_vs\_\*\_OPLS\_DA\_permutation.pdf 差异分组OPLS-DA检验图
    - \*\_vs\_\*\_OPLS\_DA\_permutation.png 差异分组OPLS-DA检验图
    - \*\_vs\_\*\_OPLS\_DA.png 差异分组OPLS-DA得分图
    - \*\_vs\_\*\_pca3D.pdf 差异分组PCA分析三维图
    - \*\_vs\_\*\_pca3D.png 差异分组PCA分析三维图
    - \*\_vs\_\*\_pca.pdf 差异分组PCA分析二维图
    - \*\_vs\_\*\_pca.png 差异分组PCA分析二维图
    - \*\_vs\_\*\_Top\_20\_FC\_change.pdf 差异分组差异倍数柱图
    - \*\_vs\_\*\_Top\_20\_FC\_change.png 差异分组差异倍数柱图
    - \*\_vs\_\*.volcano.pdf 差异分组火山图
    - \*\_vs\_\*.volcano.png 差异分组火山图
    - KEGG 差异分组KEGG注释结果
      - Graph 差异分组KEGG富集结果
      - kegg\_map 差异代谢物通路图注释
        - ko\*.html 差异代谢物通路图
        - ko\*.png 差异代谢物通路图
  - venn 各分组间的韦恩图
    - \*\_venn.png 差异分组间的韦恩图
    - All\_diff\_stat.txt 差异代谢物数目统计结果
    - Diff\_Venn\_Intersection.xls 差异代谢物交集统计结果
    - Diff\_Venn\_Union.xls 差异代谢物并集统计结果
- readme.pdf 说明文档

Copyright © 2009-2021 北京百迈客生物科技有限公司版权所有 京ICP备10042835号

公司地址：北京市顺义区南法信府前街12号顺捷大厦5层

- Tel:400-600-3186
- Fax:010-57045001
- Tel:400-600-3186
- E-mail:tech@biomarker.com.cn
- 微信:biomarker\_tech
- 百迈客生物云平台
- 关于我们
